# An estimation of traffic related CO_2_ emissions from motor vehicles in the capital city of, Iran

**DOI:** 10.1186/1735-2746-9-13

**Published:** 2012-11-28

**Authors:** Aliakbar Kakouei, Ali Vatani, Ahmed Kamal Bin Idris

**Affiliations:** 1 Chemical Engineering Department, College of Engineering, University of Tehran, Tehran, Iran; 2 Department of Petroleum and Renewable Energy Engineering, University of Technology of Malaysia (UTM), Johor, Malaysia

**Keywords:** CO_2_ emission, Vehicle, Traffic, Transportation

## Abstract

Vehicle exhaust is a major source of anthropogenic carbon dioxide (CO_2)_ in metropolitan cities. Popular community mode (buses and taxies) and about 2.4 million private cars are the main emission sources of air pollution in Tehran. A case survey has conducted to measure CO_2_ in four popular vehicles, bus, taxi, private car and motorcycle, which moved in the city with respectively 7800, 82358, 560000 and 2.4 million per day in 2012. Results indicated that the contribution of CO_2_ emissions increased in the following order: private car, motorcycle, bus and taxi. The overall average for the contribution of CO_2_ emissions in the private car, motorcycle, bus, and taxi were 26372, 1648, 1433 and 374 tons per day, respectively. Our results also showed that the urban transport operation consume an estimated 178 and 4224 million liter diesel and petrol per year, respectively, that have released about 10 million tons of CO_2_. The average contribution of CO_2_ emissions of private cars in Tehran was higher (88%) than other vehicles. It was concluded that high volume of traffic, transport consumption of fossil fuels and shortage of adequate public transport system are responsible for the high CO_2_ level in environment in Tehran. Thus, it is to be expected that CO_2_ as a greenhouse gas has risen in Tehran more than ever in the following years and this would be a matter of concern for the authorities to have a comprehensive plan to mitigate this phenomena.

## Introduction

Carbon dioxide (CO_2_) is a greenhouse gas that traps the earth’s heat and contributes to climate change (Fogarty and McCally, [1]). Global climate change has become a serious problem in the world nowadays. Reports show that the anthropogenic carbon emissions and atmospheric CO_2_ are the most significant of the greenhouse gases causing global warming. Recent investigations conducted by Mann *et al*., [2]; Tans, [3]; EIA, [4] and IPCC, [5], show that CO_2_ concentrations in the atmosphere has risen from pre-industrial levels of 280 ppm to present levels of ~380 ppm and this increase in atmospheric CO_2_ is attributed to the world’s expanding use of fossil fuels and is believed to be the primary cause of global warming. Further research shows that the 270 Giga tons of anthropogenic carbon emissions over the past 200 years would have increased CO_2_ concentrations from 280 to 380 ppm (Steven *et al*. [6]).

The relationship between transportation and air pollutants, such as CO_2,_ CO, NOx and SO_2_ has been well documented in a wide range of case studies. Reports indicate that the on-road vehicle emissions constitute the major source of atmospheric CO_2_ in urban areas. It contributes around 10% of the total global and 20% of the European atmospheric CO_2_ emissions (Metz, [7]; Nejadkoorki *et al*., [8]). In 2002, Gorham showed that CO_2_ emissions from road traffic worldwide will increase by 92% between 1990 and 2020 (Gorham, [9]; Nejadkoorki, *et al*., [8]). Traffic emission estimates have been used mostly to allow decision makers to manage carbon capture and storage (CCS) projects and local air quality effectively (Carmichael *et al*., [10]; Mensink and Cosemans, [11]; John and Michael, [12]). Although there has been a few reports of ambient air pollutants such as asbestos, PM_10_, PM_2.5_ , TSP and CO concentrations in Tehran (Kakooei and Kakooei, [13]; Ghasemkhani and Nasseri, [14]; Kakooei *et al*., [15]), there have been no reports of atmospheric CO_2_ emission in ambient air of Tehran. Given this lack of data on atmospheric CO_2_ in ambient air and the background of approximately 3.5 million motor vehicles in Tehran, an estimate of atmospheric CO_2_ was urgently needed to provide the information necessary for developing effective CCS and air quality managements. The objectives of this study were: (I) to estimate CO_2_ emissions from taxies, buses, motorcycles and private cars; (II) to compare the levels of CO_2_ by motor vehicles; (III) to obtain data that will contribute to establishing background for CO_2_ levels to refer for reference in studying the effects of the high volume of vehicular traffic in Tehran.

## Materials and methods

### Study design and subjects

This study was performed in Tehran, the capital city of Iran in 2011. There are several types of fuels used in Iran (Tehran): petrol (normal and super), diesel, CNG (compressed natural gas) and liquefied petroleum gases (LPG). According to the emission inventory study results, the pollution sources in Tehran are predominately private cars using petrol: large numbers together with poor technology and low emission standards. Only a small number of cars use CNG which are neglected in our study.

The fuel consumption unit (mainly petrol) is very high in Tehran. Since petrol produced (refined) in Iran is not sufficient for all customers, the fuel is being imported by the government from abroad. Iranian fuel conservation organization (IFCO), a subsidiary of Ministry of Oil, started to convert petrol vehicles to CNG by using bi-fuel systems, but there are not enough CNG refuel stations in Tehran. An important problem in Iran is the quality of fuel due to octane number of gasoline and sulfur content in diesel fuel. The gasoline distributed in Iran comes in two quality ranges, “Normal gasoline” (87 Octane) and “Super gasoline” (95–97 Octane). Also gasoline used in Iran is lead free. Diesel fuel distributed in Iran comes in two qualities according to the sulfur content: 500 ppm for United Bus Company of Tehran (UBCT) usage and 7,700 ppm for other heavy duty vehicles.

Public transportation in Tehran consists of taxi, bus, and underground metro and it is managed by municipality of Tehran through 3 companies: United Bus Company of Tehran for buses, Tehran Taxi Management & Supervision Organization (TTMSO) for taxis and Metro Company for the subway system.

United bus company of Tehran has several types of buses consisting of MAN, Benz, Volvo and Renault that use diesel (turbo charged and natural aspirated) or CNG as fuel. According to the statistics from UBCT by March 2010, the number of buses was 7800 (composed of all the above models) (Rashidi, [16]). There are many types of taxis in Tehran: Peugeot 405, Peykan, Hyundai, Samand (a car produced in Iran that uses Peugeot engine), a few of them are equipped with CNG system. Taxis services are in 5 types: Orange taxi, Terminal taxi, Airport and Railway station taxi and Tell taxi. Number of cars in the taxi system was 82358(TTMSO, [17]).

According to the traffic department of Tehran, the daily urban fleet in the Tehran region comprised 3.5 million vehicles (cars, buses and motorcycles) in June 2011 Rashidi [16]. Out of the total households living in Tehran, about 2.4 million (nearly 50%) have private cars and 14% have motorcycles.

### Data gathering and analysis

As mentioned above, the daily urban fleet in 22 municipality regions of Tehran comprised about 3.5 million vehicles that consist of 2.4 million private cars, 560,000 motorcycles, 78,000 buses and 82358 taxis (Table 1). A mathematical model with a macro-scale approach was used to estimate the CO_2_ emissions of each type of mentioned vehicles (Ribeiro and Balassiano, [18]). In the model, the average consumption for all of the vehicles has taken into account. It was needed also to have the average run in kilometer to calculate the total fuel consumption (liter) and the total CO2 emission (E_M_). Other required data such as specific gravity, calorific power, and emission factor of both diesel and petrol are also needed to calculate the amount of CO2 emission in the atmosphere. Briefly, the model is specified as follows:

(1) $$F_{C}=A_{C}\times V\times R_{D}$$

**Table 1:** Average fuel consumption and number of vehicles with their average running per day

**Type of vehicles**	**V**	**R**_**D**_	**A**_**V**_
		**(km)**	**(lit/km)**
Bus	7800	250	0.25
Taxi	82358	185	0.1
Private Car	2400000	43	0.1
Motorcycle	560000	40	0.04

*V, number of vehicles; R_D_, average running /day; A_V_, average consumption.

Where

F_C_ = fuel consumption (diesel or petrol) (L)

A_C_ = Average fuel consumption by each type of vehicle per kilometer (L/km)

V = Number of each type of vehicle

R_D_ = Amount of running per day by the vehicle (km)

(2) $$EM\left(CO_{2}\right)=F_{C}\times SG_{F}\times CP_{F}\times EF_{F}$$

Where:

SG_F_ = Specific gravity of the used fuel (kg/m^3^)

CP_F_ = Calorific power of the fuel (kcal/kg)

EF_F_ = Emission factor of the fuel (tco2/TJ)

In the estimate, as it is mentioned earlier, the vehicles running on CNG and other types of fuel are not taken into account.

## Results

### Potential CO_2_ emission conditions

Tehran with a day time population of some 10 million and with a metropolitan area of over 2000 km^2^ is the capital city of Iran and regional center of Tehran province. The city of Tehran is the first most populous city in Iran. The city is hemmed in by Alborz Mountains to the north, resulting in an increasing volume of pollutants such as CO_2_ and CO. Tehran’s high altitude, ranging between 1200 to 1500 meter above the sea, also makes fuel combustion inefficient, adding to the anthropogenic carbon and atmospheric CO_2_ problem (Kakooei and Kakooei, [13]). The rapid increase in population has generated an increase in urban trips (Rashidi, [16]). The increasing number of private cars in Tehran has turned into a major problem for this metropolis. It is possible to observe a high concentration of private cars (2.4 million) in the city, mainly during the peak morning and evening hours. As noted in Table 1, private cars are the most important means of transportation in the city of Tehran. The bus and taxi systems are the means of public transportation in this city, with about 90000 vehicles (Table 1). As mentioned, metro is the other mean of public transport in the city of Tehran, and its share is growing. In 1997 the Japan International Co-operation Agency (JICA) predicted that about 71% of air pollution such as CO2 and CO in Tehran is produced from mobile emissions (Kakooei *et al*., [15]).

### The contribution of buses to CO_2_ emissions

The Ac of the buses operating and R_D_ of a running per day in Tehran are given in Table 1. As summarized in this Table, the average consumptions and R_D_ of running were 0.25 L and 250 km, respectively. As noted in the methods, the total buses which are operated in the urban fleet of Tehran are 7800 vehicles. Table 2 shows the diesel emission factor, calorific power and specific gravity which have been used in the model. The results of the model are given in Table 3, which show that calculated diesel consumption by buses per day was 487,500 L. Based on the total amount of liter of diesel burnt each day, the CO_2_ emission from buses was 1433 tons per day in Tehran region.

**Table 2:** Chemical properties of fuels used in urban transportation fleet in Tehran

**Type of Fuel**	**SG**_**F**_	**CF**_**F**_	**EF**_**F**_
	**(Kg/cuM)**	**(Kcal/kg)**	**(tCO2/TJ)**
Diesel	885	10700	74.1
Petrol	737	11464	69.3

*SG_F_, specific gravity of fuel; CF_F_, calorific power of fuel; EF_F_, emission factor of fuel.

**Table 3:** Contribution of vehicles to CO2 emission with their fuel consumption

**Type of vehicles**	**F**_**C**_	**E**_**M**_**CO2**
	**(lit)**	**(tons)**
Bus	487500	1433
Taxi	152362	374
Private Car	10750000	26372
Motorcycle	672000	1648

*F_C_, total consumption/day; E_M_ CO_2_, CO_2_ emission/day.

### The contribution of taxies to CO2 emissions

Based on the results presented in Tables 2 and 3, the total consumption value in the taxies was 152,362 L, which is considerably lower than the diesel consumption by buses. Table 3, also shows the CO_2_ emission from taxies (374 tons), which was estimated for one day.

### The contribution of private cars to CO_2_ emissions

Average running (R_D_) and average consumption (Av) of private cars are given in Table 1. The private car fleet considered in the estimation is approximately 2,400,000 vehicles (Table 1). The average fuel consumption of the private car was 10.8 million L/day, which is considerably higher than other vehicles (Table 3). The contribution of private cars to CO_2_ emissions are presented in Table 3. As the results show, the total amount of CO_2_ which is released into the atmosphere by the private cars was 26,372 tones.

### The contribution of motorcycles to CO_2_ emissions

According to the number of active motorcycles moving in the city every day (560,000), the average amount of running per day (40 km) and also the average petrol consumption (0.03 L/km), the total petrol consumption by the motorcycles was 672,000 L. CO_2_ which is emitted by the motorcycles are given in Table 3. As it has been illustrated, the contribution of motorcycles in CO_2_ emission was 1648 tons per day.

## Discussion

The contribution of motor vehicles to CO_2_ emissions has been rarely estimated in Iran. As noted in the introduction, the anthropogenic carbon emissions and atmospheric CO_2_ are the most significant greenhouse gases causing global warming (Harding, [19]). Previous and current trends indicate that CO_2_ emissions from road traffic worldwide will increase by 92% between 1990 and 2020 (Gorham, [9]). As the results of Tehran showed, the urban transport operation consumes an estimated 178 and 4224 million liter diesel and petrol, respectively per year that have released about 10.9 million tons of CO_2_.

**Figure 1:**
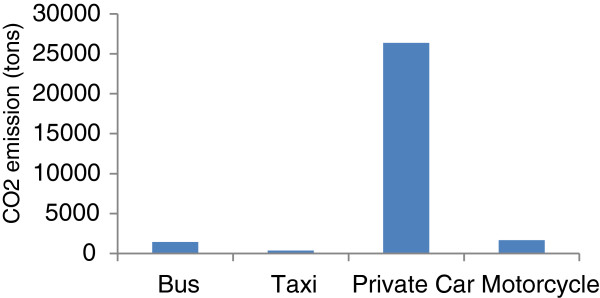
Contribution of vehicles to CO2 emission.

As shown in Table 3, about 88% of all anthropogenic CO_2_ emissions in Tehran are produced by private cars, while the public sector has lower CO_2_ emission than private vehicles. Congruent with previous studies in other regions, this research confirms that shortage of adequate public transport system is the basic responsible to higher level of CO_2_ emissions in urban areas (Samaras *et al*., [20]; Metz, [7]; Reckein *et al*., [21]). It is interesting to note that cities with a higher contribution of public transport produce less CO_2_ from public transport than cities which rely mainly on private motorized mobility (UITP, [22]). According to the Ribeiro and Balassiano, [18], daily CO_2_ emissions from buses and private cars in Rio de Janeiro were 4050 and 7947 metric tons respectively, which are lower than the amounts found in this study. In general, in the current study, the level of CO_2_ emissions from private cars (26,372 L) has discrepancy to those reported in some of the previous studies (Ribeiro and Balassiano, [18]; Metz, [7]). The origin of CO_2_ emissions among major motor vehicles in Tehran are shown in Figure 1. In comparison to the total vehicles moving in the city, the CO_2_ emissions by private cars are more heavily concentrated in transportation, which produce 88% of its total emissions, compared to 12% public transport share throughout the city.

## Conclusion

Today, Asia is a small contributor to the world’s emissions of greenhouse gases. But, Asia anthropogenic carbon emissions from transportation, mostly based on cars, are predicted to grow threefold by 2030 as both automobile ownership and motor vehicle use expand (United Nation, [23]). In conclusion, the present study strongly suggests that the high levels of CO_2_ emission are largely attributed to the lower modal share of public transport. It may also be concluded that high volume of traffic, transport consumption of fossil fuels and shortage of adequate public transport system are responsible for the high CO_2_ level in the environment of Tehran. Thus, it is to be expected that CO_2_ as a greenhouse gas has increased in Tehran in recent years and this would be a matter of concern for the authorities to predict a comprehensive plan to mitigate this phenomena.

## Competing interests

The authors interest is in CO2 related global warming, capture and sequestered it in underground formations.

## Authors’ contribution

AK, AV and AKBI: All authors read and approved the final manuscript.
